# “I wanna live in a world where change is possible”: co-designing guidance for inclusive eating, exercise, and body image psychopathology outreach resources for men

**DOI:** 10.1186/s40337-026-01562-5

**Published:** 2026-03-05

**Authors:** George Mycock, James Downs, Győző Molnár, Una Foye, Heike Bartel, Jessica R. Griffiths, Christian Edwards

**Affiliations:** 1 School of Sport and Exercise Science, University of Worcester, Henwick Grove, WR2 6AJ Worcester, UK; 2 Person with Lived Experience and Peer Researcher, Cardiff, UK; 3 Mental Health Nursing, Health Service and Population Research Department, Institute of Psychiatry, Psychology & Neuroscience, King’s College London, London, UK; 4 School of Cultures, Languages and Area Studies, University of Nottingham, Nottingham, UK; 5 South London and Maudsley NHS Foundation Trust, London, UK

**Keywords:** Eating disorders, Body dysmorphic disorder, Body image, Men, Help-seeking, Co-design, Facilitation

## Abstract

**Background:**

Men are underserved in research on eating, exercise and body image psychopathology (EEBIP), and remain underrepresented within healthcare settings despite growing clinical need. One barrier to men’s help-seeking for EEBIP-related concerns is that public-facing healthcare information/resources often appear unwelcoming to them, suggesting the need for more inclusive, gender-sensitive resources that engage men and address their specific needs. This study aimed to explore men’s perspectives on the design of inclusive EEBIP resources, to inform the iterative co-design of guidance for future resource development.

**Methods:**

This study employed a lived experience-led approach, integrating a modified nominal group technique with participatory research methods, as part of an iterative co-design of a guidance document. Six men with lived experience of EEBIP ranked their preferred features of male-inclusive resources from a broader list of content and format ideas generated through a survey of 42 men. Interview and focus group discussions followed, exploring the underlying reasons why the men with EEBIP experience believed these content and format ideas would support men’s help-seeking for EEBIP. Finally, a draft guidance document, underpinned by the results of the discussions, was designed and iteratively edited, following feedback from healthcare organisation representatives, academics, and men with lived experience.

**Results:**

Thematic analysis of the discussions identified five themes to inform the development of future resources. The five themes are titled: (1) Designing accessible resources that navigate men’s readiness; (2) Authentic voices: inclusive, conversation-led outreach; (3) Self-realisation: refraining from labelling men as ‘unhealthy’ or ‘disordered’; (4) Purpose and progress driven resources; (5) Images: the line between helpful and harmful isn’t always clear. These themes are discussed alongside EEBIP and men’s mental health help-seeking literature.

**Conclusions:**

This is the first study to centre men’s experiential knowledge to explore how healthcare organisations can facilitate men’s help-seeking for EEBIP-related concerns via public-facing resources. The themes generated in this study reflect findings of studies exploring the facilitation of men’s general mental health help-seeking, whilst contributing novel EEBIP-specific findings. The results of this study can support EEBIP healthcare organisations to develop public-facing resources that are more inclusive of men.

**Supplementary Information:**

The online version contains supplementary material available at 10.1186/s40337-026-01562-5.

## Abstract (plain language summary)

Men have previously reported that public-facing healthcare resources, from eating, exercise, and body image psychopathology (EEBIP) healthcare organisations (e.g., eating disorder services), feel unwelcoming to them. We worked with men, including men experiencing EEBIP, to identify how healthcare organisations can create more men-inclusive resources. First, men suggested ideas for the content and format of future materials via a survey. These ideas were then rank-ordered by men with EEBIP to identify their preferred options for future healthcare resources. Next, via an interview or a focus group, the men with EEBIP discussed why they preferred the selected options and how these underpinning reasons might inform future practice. From these discussions, five key considerations for future resources were developed. Future public-facing resources should: (1) be tailored to specific audiences of men, (2) reflect the authentic voices of men with lived experience, (3) encourage self-recognition of concerns rather than imposing labels, (4) demonstrate the purpose of and progression through care, and (5) balance engaging images with sensitivity. These themes were then used to co-design a guidance document for healthcare organisations, offering practical steps to ensure information about EEBIP is relevant, accessible, and supportive for men.

**Supplementary Information:**

The online version contains supplementary material available at 10.1186/s40337-026-01562-5.

## Introduction

### Eating, exercise, and/or body image psychopathology

Eating, exercise, and/or body image psychopathology (EEBIP) is an inclusive term encompassing a broad spectrum of behaviours, cognitions, and diagnoses related to body image concerns. Examples of EEBIP are: clinically recognised mental illnesses such as eating disorders (EDs), body dysmorphic disorder (BDD), and muscle dysmorphia [1]; experiences related to these mental illnesses but not yet recognised in diagnostic manuals (e.g., muscularity-oriented disordered eating, MODE [2]; and the experiences of individuals who have not received a formal diagnosis [3]. Mycock et al. [3] introduced a similar term (i.e., eating and/or body image psychopathology; EBIP) to increase recognition of the spectrum of behaviours/cognitions that men may present with, which often fall outside existing definitions of illness [4]. This paper introduces an extended version of the term used by Mycock and colleagues to include those whose primary concern relates to exercise, such as compulsive or excessive exercise behaviours [5].

### Men’s EEBIP and EEBIP help-seeking

Researchers have noted that men’s experiences of EEBIP remain underexplored, and many studies lack methodological rigour [1, 6–8]. In recent years, advancements have been made, including the development and validation of multiple measures that better capture men’s symptoms [9–12], compared to many widely used measures [6, 13–15]. Despite the development of these male-focused measures, there remain issues regarding gaps in measures for specific contexts and subgroups, concerns with comparability, and limited uptake in clinical settings (for review, see [16]).

Other advancements have been made in the form of tailored harm reduction guidance for healthcare workers regarding muscularity-oriented behaviours often seen in men [17], and calls for specific medical treatment guidance for adolescent males with EDs [18]. Yet despite these advancements, many healthcare challenges remain, as healthcare providers report lacking confidence due to limited training and evidence-based treatment options for EEBIP in men [19–21], and men report facing barriers due to stigma, language used in services, inappropriate service pathways, and poor EEBIP healthcare experiences [3, 22–24]. With this evidence in mind, it appears that men are underrepresented in clinical and academic EEBIP settings.

Considering these academic and clinical concerns, it may be unlikely that EEBIP healthcare systems can provide equal support to men compared to other genders. Alarmingly, EDs in men have risen globally [25], with the UK’s National Health Service (NHS) reporting a 128% rise in male inpatient clinical samples from 2015 to 2021 [26]. Men also have a higher ED-related risk of mortality compared to women [27, 28]. Data from the UK indicate that men account for 25–33% of ED cases [29, 30], yet NHS data show that male patients comprise only 7–10% of ED admissions [26, 31]. Similar disparities are seen in other forms of EEBIP. For instance, Veale et al. [32] found that while adult community samples show 21% more female cases of BDD compared to males, inpatient settings report 71% more female than male cases, and community healthcare services report 41% more female cases compared to males. These findings may suggest that men’s access to care is limited in ways that are not explained by prevalence alone.

### Organisational barriers: a lack of men-inclusive resources

A recent review [3] found multiple reports of organisation-level barriers to EBIP help-seeking (i.e., barriers stemming from healthcare systems and services) that remain under-researched. Indeed, studies share men’s reflections of limited help-seeking due to the content of EEBIP healthcare resources and information, which left men feeling unrecognised and misunderstood [24, 33–36]. These concerns with a lack of men-inclusive resources suggest that the development of new or existing public-facing resources may improve men’s help-seeking for EEBIP care. Accordingly, evidence from the wider men’s mental health field also reinforces the need for inclusive public-facing resources that support men’s help-seeking [37]. Sheikh et al. [38] recently reviewed the literature on barriers and facilitators to help-seeking for mental health concerns in young men and boys, identifying “male-friendly campaigns” (p. 575) as the only consistently reported facilitator across the literature. This theme was derived from five peer-reviewed studies that focused on increasing initial help-seeking through public-facing information [37, 39–42]. The studies covered a range of male populations (e.g., different ages, ethnicities, and locations), and types of support (e.g., university services, informal networks, and formal healthcare), but focused mostly on general mental health concerns (e.g., anxiety), with no references to EEBIP.

### Learning from men: how to better facilitate men’s EEBIP help-seeking

The facilitators highlighted in these studies speak to the need for lived-experience-led outreach information that uses non-medicalised, solution-focused, and male-friendly language, which clarifies who can access and how to access care, and is delivered by relatable and relevant public figures [37, 39–42]. Additional research may build on this knowledge by further exploring men’s ideas on how to facilitate men’s help-seeking via public-facing information. Research can also look to corroborate and contrast the appropriateness of the suggested facilitators across different groups of men. As little is known about the facilitators of men’s EEBIP help-seeking [3], research may look to investigate men with lived experience of EEBIP to learn from men’s experiential knowledge of (potential) facilitators of EEBIP help-seeking. To help tap into and centralise men’s experiential knowledge, research may look to build on the growing body of co-production/co-design research to develop facilitation-focused information to inform future resource development [43–48]. These insights would, in turn, support healthcare organisations in producing resources that are more welcoming, inclusive, and responsive to men’s diverse needs.

### Aim

This study aimed to explore men’s perspectives on the preferred format and content of public-facing resources that may facilitate men’s EEBIP help-seeking. This exploration then informed an iterative co-design of guidance for EEBIP healthcare organisations. In this paper, we report and discuss men’s perspectives on future resources and describe the methods used during the iterative co-design of a guidance document. The resulting guidance document can be found as an additional file for readers to consider for implementation.

## Methods

### Participants

Individuals who took part only in the idea-generation survey of Phase 1 (detailed below) are referred to as participants. Participants who self-identified with lived experience of EEBIP. and opted to engage in the study further, were then referred to as Lived Experience Partners (LEPs). All participants and LEPs self-identified as men.

Forty-one men (mean age = 31.6 years, SD = 11.1) completed the Phase 1 idea generation survey. Of those 41 men, six opted in to take part as LEPs (mean age = 34.2 years, SD = 8.1), completing a ranking survey and being involved in Phases 2 and 3. Participant and LEP demographics are in Tables 1 and 2.

**Table 1:** Participant demographics

Demographics of participants	Frequency	Percentage
*Gender*		
Cisgender man	38	92.7
Transgender man	1	2.4
Prefer not to say	1	2.4
Prefer to describe	1	2.4
*Ethnicity*		
White British	34	82.9
Asian/Asian British	3	7.3
Black/African/Caribbean/Black British	3	7.3
Other Ethnic Group	1	2.4
*Sexual orientation*		
Heterosexual/straight	28	68.3
Gay	11	26.8
Queer	1	2.4
Prefer not to say	1	2.4
*Lived experience of EEBIP?*		
Yes	24	58.5
No	17	41.5
*Orientation of EEBIP*		
Thinness-oriented only	6	14.6
Muscularity-oriented only	7	17.1
Thinness and muscularity	6	14.6
Muscularity and ‘other’	1	2.4
Other	1	2.4
Prefer not to say	3	7.3
None	17	41.5
*Previously sought help for EEBIP*		
Yes	10	24.4
No	14	34.1
Do not experience EEBIP	17	41.5

All responses were generated via multiple-choice questions

**Table 2:** Demographics of LEPs

LEP code	Gender	Ethnicity	Age	Sexual orientation	Orientation of EEBIP	Previously sought help for EEBIP (yes/no)
LEP1	Cis-man	White British	37	Homosexual	Muscularity only	No
LEP2	Cis-man	White British	36	Heterosexual	Thinness and muscularity	No
LEP3	Cis-man	Asian/Asian British	26	Heterosexual	Thinness only	Yes
LEP4	Cis-man	White British	27	Heterosexual	Muscularity only	No
LEP5	Cis-man	White British	48	Homosexual	Muscularity only	No
LEP6	Cis-man	Other (White American)	31	Homosexual	Thinness and muscularity	Yes

### Ethical considerations

The University of Worcester Health and Science Research Ethics Panel (HS24250016-R) granted ethical approval, and participants provided written informed consent before each phase of data collection. Within the participant information sheet, referral information was provided to participants, containing information on support available and details of relevant support organisations. Data were collected, stored, and handled in accordance with institutional data protection guidelines and UK GDPR requirements. No directly identifying information was collected from participants. All data were anonymised during transcription and prior to analysis. Data were stored on password-protected, encrypted university servers with access restricted to the named research team members only. Audio recordings were destroyed following transcription and verification.

### Study design

**Fig. 1:**
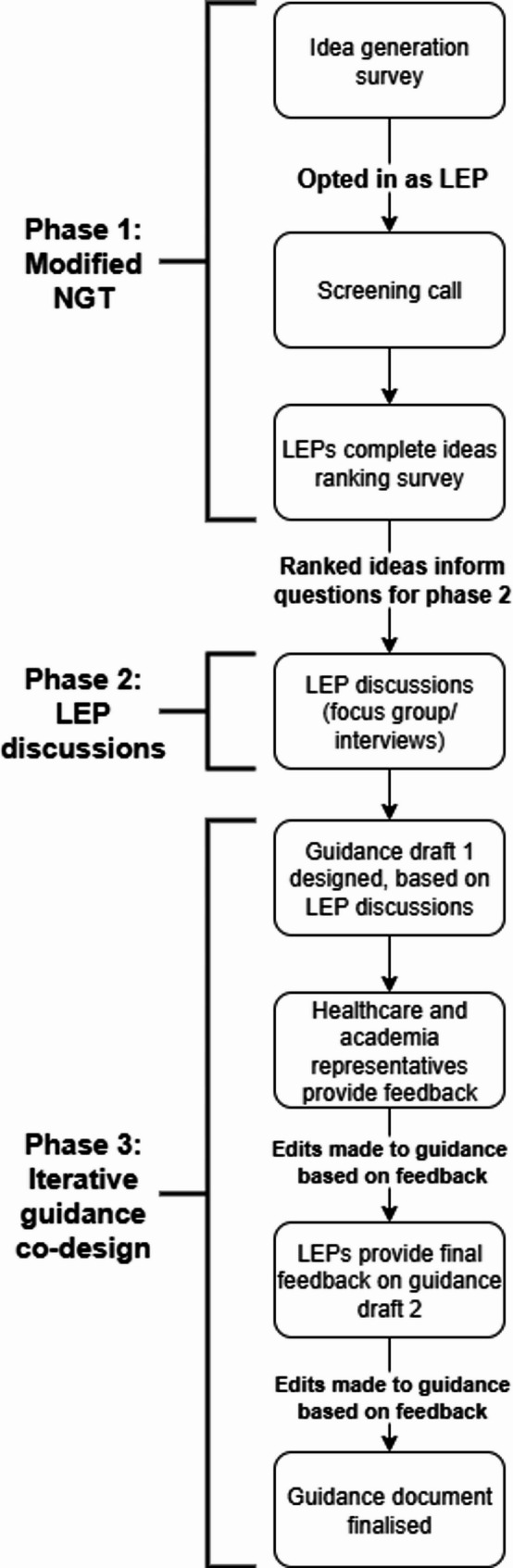
An illustrative perspective of the present study design. *NGT* nominal group technique, *LEP* lived experience partner

This study followed a mixed-method methodology that combines consensus-generation via a nominal group technique (NGT; see Table 3, [49]), qualitative exploration via interviews and focus groups, and an iterative co-design method (see Fig. 1). The study is grounded in principles of co-production by prioritising experiential knowledge of men with lived experience, and aiming to balance power relations between the researchers and men with lived experience where possible [47, 48, 50]. Despite following these co-production principles, lived experience perspectives were not involved in the selection of the study aim, and therefore the project is more accurately described as co-design [51]. In line with the focus on co-design/co-production, methods were selected to access and centre the experiential knowledge of men through a dialogical and iterative approach.

This design was rooted in a critical realist paradigmatic perspective; this perspective combines realist ontology with the epistemological view that knowledge is constructed. Through this paradigm, the researchers assume that problems are ‘real’ and seek evidence-based solutions, while acknowledging that knowledge is socially constructed and may not apply to all scenarios [52, 53]. This paradigmatic perspective aligns with the dialogical and iterative methods of the present study, as researchers aimed to construct new knowledge of how to effectively facilitate men’s EEBIP help-seeking—working alongside men with experiential understanding—through phased discussion and co-design of a guidance document. The researchers also took a value-inclusive axiological stance, acknowledging the role of the researchers’ values and experiences in the construction of knowledge.

### Phase 1: modified nominal group technique

An NGT design was used in phase 1 of this study to identify priority resource formats and content ideas, suggested by men, to inform the questions for phase 2 discussions with lived experience partners (LEPs). NGT is a structured, multi-step method that combines individual idea generation with group discussions in which ideas and/or themes are prioritised [54]. In this study, NGT was modified to consist of an online stepped approach where the ideas suggested by the initial group (i.e., adult men) were then rank-ordered and prioritised by LEPs rather than the entire group (see Table 3, [49]). The top-ranked ideas from the NGT (presented in the results section; see Tables 4 and 5) were added as prompts to the questions asked in phase 2 (although conversation was not restricted to only discussing these ideas). The various stages of the NGT took place online to increase accessibility for men and reduce peer influence during the ranking of ideas.

**Table 3:** Modified NGT methods

NGT stages [49]	Modified NGT stages of the current study
Introduction—where the facilitator introduces the purpose of the meeting	Participants were introduced to the purpose of the survey within the survey
Individual responses—where each individual provides separate responses to set questions	The idea generation survey asked participants to list ideas for the format and content of EEBIP outreach resources that can engage men
Clarification and consolidation—where responses are read out and clarified one by one by participants, then similar/same items are merged under one response	The lead researcher merged similar ideas together, resulting in 16 unique format ideas and 30 unique content ideas, which were presented to LEPs at the next stage
Ranking responses—where participants rank their top responses individually in order of importance	LEPs chose their first, second, and third favourite format and content ideas via an online ranking survey

### Phase 2: participatory methods

Participatory methods were undertaken with LEPs in phase 2 to offer various ways to discuss LEPs’ perspectives of outreach resources for men. Consistent with co-production principles, participatory methods were used to allow LEPs to choose the data collection method that best suited them [47, 48, 50]. LEPs selected either the 1 to 1 semi-structured interview or the focus group options. Through their chosen method, LEPs discussed the top-ranked content and format ideas from Phase 1, the reasons why they think these ideas would or would not effectively reach men and improve help-seeking, and any additional factors that they felt must be considered when developing public-facing EEBIP resources for men.

### Phase 3: iterative co-design

 George Mycock (GMy) and James Downs (JD) led the iterative co-design process, underpinning the initial guidance document draft with the themes from Phase 2. This iterative process, detailed in the procedure section, involved drafting, external feedback, and LEP member checking [55] to refine the guidance.

### Procedure

#### Phase 1

The online idea-generation survey was distributed via the social media platforms (Instagram, X, Facebook, and LinkedIn) of the authors of this paper and emailed to undergraduate cohorts in the UK over a period of 4 weeks. Advertisements asked for those who fit the inclusion criteria (i.e., identify as men and are 18 years old and above) to help “create guidelines to inform future resources to help men get help” for EEBIP. No incentives were provided beyond the potential to impact future resources to support men. The survey contained 11 items that asked participants to confirm they fit the inclusion criteria, and provide demographic information, including age, ethnicity, sexual orientation, EEBIP personal experience, and whether they had sought help for EEBIP previously. Regarding EEBIP experience, the survey asked, “Do you experience or have you experienced any variation of eating, exercise, and/or body image concerns?” before asking participants to provide further details and indicate whether these concerns were oriented toward muscularity, thinness, both, or other via a multiple-choice question. Following demographic questions, participants were presented with two open questions that asked for participants’ ideas of potential formats and content for future resources. It took participants approximately 8 min to complete the survey. Responses were extracted to a Microsoft Excel file for descriptive analysis. Open question responses listing ideas for content and format were extracted to a Word document. GMy then reviewed these format and content ideas, removed duplicates and uploaded the remaining responses to the ranking survey (see next paragraph). The final page of the survey listed LEP inclusion criteria (i.e., lived experience of EEBIP and able to use Microsoft Teams for a short screening call), and participants were given the option to opt in as an LEP.

LEPs were contacted by the lead researcher who presented an additional participant information sheet and arranged a screening call. This study used screening calls to ensure the authenticity of LEPs due to the recent surge of false participants [56]. Eligible LEPs (*n* = 6) provided consent for the remaining steps of phase 1 and for involvement in phases 2 and 3. LEPs completed a ranking survey where they ranked their top three format and content ideas, based on which they perceived as the most effective at reaching men and facilitating help-seeking, before selecting their preferred Phase 2 participation method. The ranking survey provided a list of 16 unique format ideas and 30 unique content ideas (see Tables 4 and 5) for the LEPs to rank. A points-based analysis system (described below) identified the highest-ranked format and content ideas, based on LEPs’ top three preferences.

#### Phase 2

LEPs were contacted by the lead researcher to arrange individual interviews or focus groups via Microsoft Teams (three LEPs selected the interview and three selected the focus group). The three interviews (facilitated by GMy; lasting 62, 67, and 81 min) and the focus group (facilitated by both GMy and JD; lasting 117 min) followed the same set of questions, which were informed by the ranking survey from Phase 1. The questions prompted LEPs to discuss what they liked/disliked about the top-ranked ideas, before being asked to discuss the use of images, language, and information in EEBIP outreach resources for men. The focus group was co-facilitated to enhance rapport building and offer an additional researcher’s perspective. Interviews were not co-facilitated, as the researchers felt the imbalance of researcher to LEP (i.e., two researchers and one LEP, if co-facilitated) may have emphasised power imbalances [57]. Throughout each session, participants could ask questions and, in the focus group, contribute to shared communication guidelines.

#### Phase 3

Following analysis of Phase 2 discussions (analysis outlined below) GMy, with assistance from JD, designed an initial draft of a guidance document, informed by the themes from Phase 2. The first draft was subsequently sent to representatives from academia (*n* = 5), EEBIP healthcare services (*n* = 6), and EEBIP charities (*n* = 4), who were invited to provide feedback on the clarity of the content and its practical utility. GMy and JD then made adjustments to the guidance draft based on this feedback. The second draft was then sent out to the LEPs for member checking [55], where LEPs were asked to fill out an anonymous survey which asked them to highlight areas of strength, areas in need of improvement, and anything they deemed missing and/or needing to change. This LEP feedback was then used to inform the final edits made by GMy and JD, resulting in the final guidance document (see additional file 1).

### Data analysis

The Phase 1 survey had no missing data. As noted above, a points-based system was used to analyse the ranking survey in phase 1 (i.e., 1 st = 3 points, 2nd = 2 points, 3rd = 1 point). This analysis was selected to mimic the in-person ranking of ideas present in ‘typical’ NGT [49], to reflect LEPs’ preferred content and format options.

The transcripts from phase 2 were analysed using experiential abductive reflexive thematic analysis (RTA; [58, 59], to identify themes of the LEP discussions which would inform the initial iteration of the guidance document. RTA recognises the researchers’ active role in meaning-making as opposed to other forms of thematic analysis [60, 61]. This analytical method was chosen as it aligns with the epistemological and axiological perspectives of the study. RTA was guided by the six phases of thematic analysis provided by Braun and Clark [60, 62–64]. First, GMy transcribed the recordings and both GMy and JD familiarised themselves with the data by reading the transcripts. Both independently coded the transcripts, identifying meaningful units of text, before clustering the coded units of text into interpretive themes [63]. With RTA, a second coder is not required due to the acceptance of axiological input; however, JD offered critical reflexive input and an alternative perspective during GMy’s refinement of the themes, before beginning the write-up. The wider research team also provided critical discussion of themes to promote further reflexivity from GMy.

### Positionality

As this study took a value-inclusive axiological stance, reflected in the analytical method used (i.e., RTA). The inclusion of researcher positionality is vital to provide a transparent perspective on how researchers’ identities, experiences, and perspectives may have influenced the research process [65]. Whilst including positionality, the researchers also acknowledge that the findings of the present study speak to the researcher’s interpretation, and do not account for all possible interpretations of the data.

Two researchers were primarily involved in the data collection and analysis of the present study (GMy and JD). GMy is a 29-year-old white, heterosexual, cisgender man with lived experience of help-seeking for muscularity/thinness-oriented EEBIP. His research background is primarily in quantitative research. JD is a 35-year-old white, homosexual, cisgender man with lived experience of multiple psychiatric, physical, and neurodivergent conditions, including a longstanding eating disorder. He has expertise in qualitative methods, including RTA.

Both researchers drew on lived experience during data collection, reflecting on their own experiences during discussions. This insider position helped build rapport and enrich discussions, but risked power imbalances. To mitigate imbalances, GMy and JD reflected before sessions and followed preset experiential involvement guidelines: only contributing their own experiences when building on topics raised by LEPs. This helped centre LEPs’ voices and reduced undue researcher influence.

The lived EEBIP experiences of GMy and JD, as men and having sought help for EEBIP in the past, will likely have influenced what they interpreted as themes during analysis, which readers should consider when interpreting the current paper. All authors also have direct and/or indirect experiences with EEBIP (e.g., excessive exercise, muscularity-oriented preoccupations, and/or disordered eating), and each brought these experiences to support GMy in shaping the study and interpreting the findings.

## Results

### Phase 1—NGT results

Forty-one participants generated 16 format and 30 content ideas. Podcasts, social media campaigns, and lived experience talks ranked highest for format, while top content priorities were information about EEBIP, reassurance that it is acceptable to struggle, and non-medicalised information (see Tables 4 and 5).

**Table 4:** Ranked format ideas

Format option	Number of 1 st place votes (3 points)	Number of 2nd place votes (2 points)	Number of 3rd place votes (1 point)	Total points
*Podcasts (could be shared via QR codes in public places leading to the podcast)	1	2	0	7
*Social media (videos, posts, campaigns)	1	1	2	7
*Lived experience talks	1	1	0	5
*Leaflets and posters	1	0	2	5
Shared via male role models (videos and quotes)	1	0	1	4
Social media ads	1	0	0	3
News stories or articles	0	1	0	2
Email campaigns from local businesses (e.g., gyms emailing information to clients)	0	1	0	2
Messages or images on gym or sports equipment	0	0	1	1

Only ideas which received a vote are included. *included in Phase 2 discussion questions

**Table 5:** Ranked content ideas

Content option	Number of 1 st place votes (3 points)	Number of 2nd place votes (2 points)	Number of 3rd place votes (1 point)	Total points
*Information about what EEBIP is (including a wide variety of men’s experiences, e.g. muscularity concerns as well as thinness concerns)	2	0	0	6
*Share that it’s okay to struggle with EEBIP (you are not less than, or less of a man)	1	1	1	6
*Non-medicalised information (less use of medical jargon, do not diagnose those engaging with the content)	1	1	0	5
Using combative recovery language (fighting against rather than recovering from)	1	0	0	3
Normalising men’s experiences (videos of the day-to-day lives of men experiencing EEBIP, prevalence stats)	1	0	0	3
Information about how to eat and exercise in a healthy way (Instead of only labelling what isn’t healthy)	0	1	1	3
Move away from BMI as a measure of severity	0	1	0	2
Informal and conversational language	0	1	0	2
Education for others (not just the men themselves, e.g., clinicians and teachers)	0	0	2	2
Information focuses on next steps to recovery (actionable information, i.e. steps to getting better from professionals)	0	0	2	2
Designed alongside men with lived experience (Make sure men with lived experience are checking/editing content before use)	0	1	0	2

Only ideas which received a vote are included. *included in Phase 2 discussion questions

### Phase 2—participatory method discussion

**Fig. 2:**
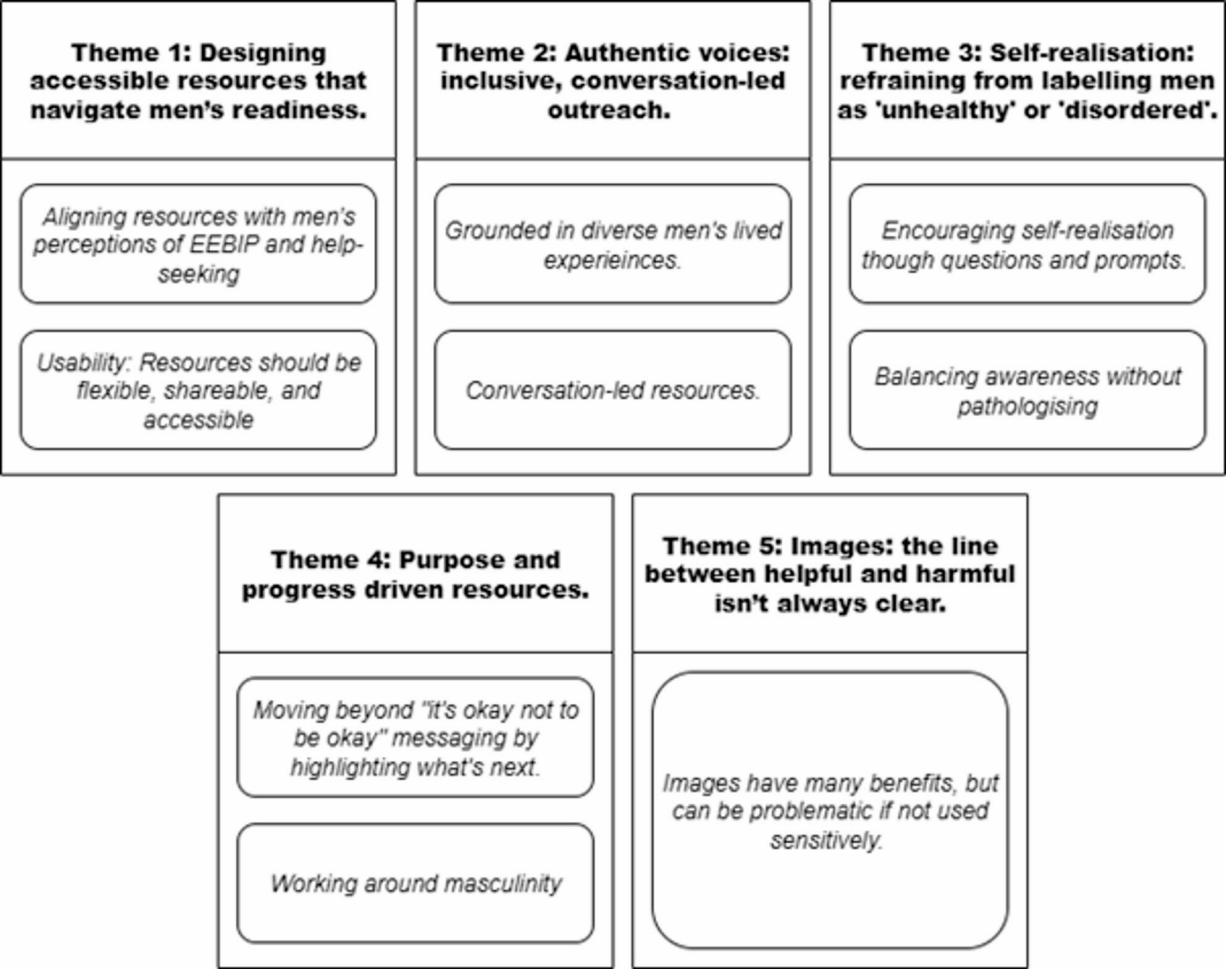
Themes and subthemes from discussions with LEPs

Five themes were developed from the RTA, each containing explanatory subthemes (see Fig. 2). These themes and subthemes are presented below.

#### Theme 1: designing accessible resources that navigate men’s readiness

LEPs stressed tailoring resources to men’s differing stages of readiness to seek help. Readiness encompassed men’s awareness/perceptions of EEBIP and preparedness to seek help, as LEPs emphasised designing content and selecting formats specific to men at various stages of readiness.

#### Theme 1—Subtheme A: aligning resources with men’s perceptions of EEBIP and help-seeking

LEPs stressed that men will have diverse awareness, attitudes, and perceptions of EEBIP and help-seeking, and that resources will therefore need to be tailored for distinct audiences of men. These might range from men who are unaware that they have a difficulty to those already considering seeking help. LEP5 reflected *“I think (tailoring resources to different groups) is interesting because there’s some people who won’t know they have… a disordered attitude… and people who do. And I think they’re very different groups of people.”*

LEPs described how different formats of resources suit men at different levels of EEBIP awareness and varying stages toward help-seeking. LEP1 highlighted how podcasts and social media can differ from other EEBIP resources as they have “*potential to be preventative”* Rather than “*reactive at the point of issue”.* LEP1 went on to explain how resources can reach people earlier if they use formats that are “*dedicated to being more forward-facing”*. LEP4 mentioned the stages of change model [66], which refers to an individual’s willingness to change a behaviour. They noted how different formats and content may be “*more likely to [reach] certain audiences at… different stages of change” (LEP4)* and how those designing materials must begin *“thinking about where that target group would be and the type of material they consume” (LEP4)*.

LEPs also identified how men’s level of readiness can influence the type of information which could deter them from seeking help. LEPs discussed how medical terminology and overly detailed descriptions of treatment, when at earlier stages of readiness, may be off-putting for some men. LEP2, for example, described how detailed information, at the initial stages of seeking or enquiring about help, can cause men to “*feel like ‘god …this is serious (…) maybe I’ll put it to the back of my mind’ or ‘actually*,* I won’t take this further’”*. LEP2 suggested that initial information should instead focus on being more “relatable” to men at those earlier stages (see themes 2 and 3).

#### Theme 1—Subtheme B: usability: resources should be flexible, shareable, and accessible

LEP discussions focused on usability features, which consisted of detailed aspects of potential resource formats that accommodate different levels and types of engagement from men. One example of resource usability was flexible engagement, as LEPs discussed how men will have different levels of interest in EEBIP information. LEPs noted that information could be offered incrementally to introduce key ideas and offer finer details through links and signposting. LEP5 reflected: *“I like lots of low-level detail. Other people will just want the headlines… incremental works… a leaflet with one or two points on it*,* but then you have a QR code that takes you to a website”*.

LEPs also noted how offering various ways to access information can help provide a wider range of men with opportunities to engage. Podcasts were highlighted as highly accessible, allowing engagement as a secondary task (e.g., listening while doing other activities). LEP3 expanded on the value of secondary task accessibility, noting that podcasts can take advantage of living in “*the most unconscious era ever because we’re on autopilot constantly”*. Podcasts were further praised for privacy (e.g., listening with headphones). LEP4 noted how *“…if someone’s feeling shame […] picking [a leaflet] up might be something that’s too difficult […] in comparison to the privacy of a podcast”.*

While privacy can support engagement, LEPs also valued open and shareable formats to help men connect and raise awareness when ready. Online resources were viewed as useful for sharing experiences and signalling the need for support. As LEP2 reflected: *“I’ve recommended [podcasts] to other people… You can really share them and hopefully that’s a sign [of needing support] as well.”*

### Theme 2: authentic voices: inclusive, conversation-led outreach

This theme addresses the value of grounding outreach in conversations with diverse men to promote authenticity and relatability.

#### Theme 2—Subtheme A: grounded in diverse men’s lived experiences

LEPs emphasised the importance of including varied voices to make resources feel authentic and challenge societal and clinical stereotypes surrounding EEBIP (i.e., skinny, white, affluent, girls; [67]). LEP3 noted that medical and societal assumptions *“bounce off each other”* reinforcing narrow ideas about who experiences EEBIP and how.

LEPs stressed the need to acknowledge muscularity-oriented experiences (e.g., muscle dysmorphia, MODE) given their link with masculine identities. As LEP5 observed, *“I don’t think that resources exist for muscle dysmorphia*,* it’s certainly not as accessible [as resources for other forms of EEBIP].”* However, LEPs cautioned that diverse representation must align with healthcare inclusivity. LEP3 gave an example, explaining that public information which *“says men can have muscularity body issues”* can lead to frustration and doubt if those who feel recognised by the inclusive resources are then told they *“don’t have an eating disorder”* and are excluded from care.

Despite the importance of balancing inclusivity of public-facing information and healthcare inclusion/exclusion policy, the LEPs consistently agreed that sharing a range of stories can help reduce fear that EEBIP healthcare is not designed for men. LEP4 reflected that diverse experiences help reinforce that *“it’s irrelevant what you look like”* and may encourage men to feel *“worthy of learning some more about [their EEBIP experiences].”*

The topic of diversity within the LEPs was a central feature of focus group discussions. LEP6 noted how they “*[found] it really interesting how [being an LGBTQ + man] wasn’t necessarily a part of the conversation [in the focus group]”*, despite the LEP sample including gay men. LEP5 noted how each of the LEPs in the focus group had high levels of literacy, stating *“we’re all really articulate on this call (…) we’re quite self-aware”*. LEP5 went on to add that this high level of literacy may mean the focus groups’ experiences and ideas are not reflective of *“[men] from different educational backgrounds [or] social backgrounds”*. These points highlight how intersectional identities, both present in LEP discussions and not, may require further exploration in the future.

#### Theme 2—Subtheme B: conversation-led resources

LEPs suggested creating resources that have conversation at the foundation, as conversation-led resources can be more engaging and authentic. LEP4 noted how inauthenticity can become a primary issue if “*trying to [reach] another culture”* via resources, as “*you’re probably gonna miss the mark”.* LEPs felt that healthcare staff should refrain from creating conversation-style resources if unable to involve and centre men with lived experience in the creation. LEP4 stated that creating conversation-style resources without lived-experience input was *“probably the worst-case scenario”*.

Most LEPs preferred conversation, stories, and narrative-focused resources over statistics and medical jargon (although offering incremental access is best; see Theme 1). LEPs described how statistics can feel separate from personal experiences, whereas an individual narrative may help men relate to the information, as LEP1 describes: *“I think it ends up being like a data thing to be like*,* ‘you’re not alone. 70% of men feel like this’*,* but actually it’s a real personal thing*,* right? (…) the hook needs to be like somebody just being like*,* ‘oh*,* for years I didn’t do this thing. For years*,* I didn’t do this thing that you are [not] doing right now.’”*.

LEPs felt that narrative and conversation-centred content would be best shared via podcasts (i.e., recorded conversations). LEPs described that podcasts enable long-form, real-world conversations that can normalise EEBIP through role models’ experiences. LEP5 shared how podcasts involving potential role models (e.g., men with lived experience) can help men adjust their perspective of EEBIP as something they could be experiencing and could seek help for: “*I think that one of the things that works well about [podcasts]*,* especially if it’s some sort of aspirational role model*,* talking about these issues… you can see that ‘if it affects them*,* then it can affect me’.” (LEP5)*.

Conversation-driven resources, with relatable role models, can also help men recognise their concerns by contextualising EEBIP within real-world examples, integrated into recognisable scenarios that draw attention to how EEBIP influences various aspects of their lives. For example, LEP3 noted how long-form conversation resources (e.g., podcasts) can help men reflect on key questions like “*what does it mean to have eating or body image difficulties and go to work?”* and how *“podcasts give you the space to make those links”.*

### Theme 3: Self-realisation: refraining from labelling men as ‘unhealthy’ or ‘disordered’

In this theme, LEPs highlight the importance of facilitating men’s own realisation and understanding of their experiences, rather than labelling behaviours as disordered.

#### Theme 3—Subtheme A: encouraging self-realisation through questions and prompts

Many LEPs noted that men often do not initially recognise their behaviours and cognitions as EEBIP-related. As such, they suggested using open-ended questions and relatable narratives to encourage self-reflection. This approach was perceived as more effective than presenting men with diagnostic labels or predefined concerns. For example, LEPs recommended asking reflective prompts such as: *“Do you often check your body in the mirror?”* or *“Do you spend a lot of time thinking about your body?”*. LEP4 noted how these prompting questions can “*lead to a sort of ‘aha’ moment”* by encouraging men to reflect on how their behaviours influence their lives.

Additionally, LEPs noted how following the guidance of theme 2 (i.e., conversation-led resources about diverse experiences) may also encourage self-realisation by recognising similar experiences to their own as examples of behaviours/symptoms that can be supported via the healthcare organisation. LEP1, for example, said *“I want to see individuals sharing similar thoughts that I’ve had*,* back to me… I don’t want like ‘70% of men feel like this’… I want it to be like ‘When I go on holiday*,* I don’t take my top off when I’m at the pool.’*

#### Theme 3—Subtheme B: balancing awareness without pathologising

While some LEPs saw value in having diagnostic criteria listed somewhere within resources, the majority cautioned against labelling behaviours as disordered or as examples of a diagnosis. LEP3 urged those designing resources to *“not diagnose people with content [of resources] because I think there’s a lot of content that does…”.* The concept of ‘diagnosing’ men via resources seemed to speak to feelings of being labelled, which may lead to fear of stigmatisation and hinder help-seeking at earlier stages of men’s readiness (see theme 1). LEP1 spoke to this, saying: *“I’ve got a shit ton of issues*,* man. I don’t want somebody throwing a poster at me telling me that I’ve got another one… I also just think it isn’t useful at the point in which we’re engaging with [outreach resources]*,* right?”.*

LEPs highlighted how societal views of EEBIP-related behaviours (e.g., regular exercise and dietary tracking) as healthy complicate outreach messaging and require consideration. Labelling these behaviours as problematic may provoke resistance, particularly among men in fitness contexts. LEP5 captured this challenge by comparing communicating EEBIP information vs. cancer information: *“Everyone wants to cure cancer. There’s not a single person who doesn’t. But dysmorphia issues? E-E-B-I-P? It’s hard to sell.”* Indeed, LEPs observed that public acceptance of health-related messaging may be limited in the context of EEBIP, as initiatives such as the Health At Every Size movement (which emphasises nuance in the relationship between body mass index, body size, and health/healthcare; [68]) can *“become quite polarising”* (LEP4) when their messages appear to contradict dominant societal norms of health. This controversy was considered to be useful at times, as a means to draw attention to the issue, but it also adds complexity, which may be circumvented if utilising self-realisation methods instead.

LEPs also noted that information listing symptoms/markers, particularly regarding body mass index and weight, may unintentionally provide some men with a false sense of health, despite experiencing EEBIP symptoms. LEP2 reflected on an example they had seen recently from a healthcare service which was “very weight focused”, which they felt may be misconstrued by men who falsely assume they do not need support due to weight gain: *“It’s like*,* ‘oh once you’re weight restored*,* then you’re OK’ but you’re not*,* are you?” (LEP2)*.

### Theme 4: purpose and progress driven resources

This theme explores how outreach content should balance the need for emotional validation with messages that support action, change, and purpose, especially in relation to masculine identity.

#### Theme 4—Subtheme A: moving beyond “it’s okay not to be okay” messaging by highlighting what’s next

Although survey responses ranked “It’s okay to struggle with EEBIP” content highly, LEP discussions revealed scepticism toward such messaging. LEP4 argued that “*I’m not much of a fan of the old: ‘It’s okay not to be okay’*,* I don’t think it’s very actionable… I think we’re probably*,* as a society*,* sort of maybe past that messaging.”* Indeed, LEPs believed that while this message has value in promoting *“vulnerability”* (LEP2), it lacked practical use and could be seen as passive or demotivating. This aversion to passivity seemed centred around the drive for change and progression, rather than learning to cope with concerns, as expressed by LEP1: *“I wanna live in a world where change is possible. Rather than like*,* coping.”.*

LEPs suggested supplementing or replacing these ‘coping’ messages with narratives that inspire change and progression. For example, LEP1 said “*I want something that’s like*,* you know*,* ‘build resilience’. That’s aspirational. Hopeful. That’s where I want my mindset to be.”*, and LEP2 noted that *“[resources can say] struggling is OK*,* but you need to get it looked at or you need to be able to speak about it.”* Framing support-seeking as a proactive and strength-based step toward personal growth was seen as more appropriate, engaging, and encouraging for men.

#### Theme 4—Subtheme B: working around masculinity

The role of masculinity was a recurring topic, particularly in relation to barriers to help-seeking. When discussing the role of masculinity, LEP3 noted: *“I think there’s potentially that feeling of ‘I don’t want to feel like I’m being coddled here’ because there’s a relationship… sometimes between awareness and almost that distorted view of compassion*,* ‘compassion is weakness’*,* right?”* This framing of compassion/support as a sign of weakness, alongside masculine pressures to not appear to be struggling, were discussed as key issues that public-facing materials needed to wrestle with.

LEPs also discussed how, for some men, muscularity-oriented EEBIP behaviours are seen as a way to obtain and sustain masculine identity and may feel like men’s only meaning/purpose in their lives. LEPs discussed how outreach resources may consider addressing these concerns by providing alternative examples of masculine identity, as LEP5 put it: *“if you want someone to dissociate muscularity from masculinity*,* you have to create a new association for masculinity. What does healthy masculinity look like?”*

LEPs also discussed the use of masculine-oriented language in resources, noting how language choices can shift men’s perspective of seeking support. LEP1 suggested that using active, rather than passive, language can help messaging appear more masculine to men: *“There’s something that feels feminine about the idea of ‘it’s OK to struggle’… and there’s something masculine about ‘building resilience’.”* LEP4 suggested compassion-focused therapy as an example of masculine terminology being used within mental health healthcare: *“one thing I really love about compassion-focused therapy… look at the three core parts ‘strength*,* wisdom and commitment’*,* they don’t sound as fluffy [as other therapy options].”*

### Theme 5—Images: the line between helpful and harmful isn’t always clear

Images (e.g., photographs, illustrations, diagrams) were identified as a particularly complex area of outreach resource development. While LEPs generally agreed on the importance of visual diversity and representation, they also expressed differing views on the use of images of bodies, particularly muscular physiques.

LEPs acknowledged the risk of perpetuating EEBIP concerns through unrealistic or triggering images. However, some argued that showing relatable images (even of bodies) may be effective for engaging men who are currently disengaged or feel unrepresented in existing materials. For example, LEP5 discussed how bodies seen on the cover of magazines such as Mens Health could serve a purpose in raising awareness of EEBIP: *“…actually*,* they’re [images of muscular men] the kind of images that are going to catch the eye of the kind of men who are struggling with this… and maybe you do need to be targeting these different communities.”*

Some LEPs also argued that the sheer lack of representation of males within EEBIP discourse may justify pictorial examples of men, even extreme examples, to raise awareness of EEBIP in men. LEP6 argued for the use of images of men’s bodies, even extreme examples, because *“imagery and representation is so important”* to help men (and men’s family/peers) to *“recognise whether [they are] on this far end of the spectrum and living with anorexia” (LEP6).*

LEPs also noted that society often glorifies even extreme muscularity as a symbol of health, which differs from the typical framing of extreme thinness in society. LEPs argued that appropriate use of muscular body images, with contextual explanation, could help open dialogue about the risks of EEBIP, rather than reinforcing them. LEP4 gave an example from their own work, providing psychoeducation within schools: *“I put up a picture of Men’s Health magazine with Zac Effron on and I go ‘who would like to look like this?’… Then I put the next slide on*,* which has a quote from him talking about how miserable he was trying to get to look like this… That hits home with a lot of the pupils.”*

Despite these arguments about the usefulness of bodily images, LEPs were adamant about the importance of sensitivity. LEP4 added that *“the images stuff has got to be so*,* so*,* so sensitive and I don’t think you just want one specific look on a poster.”* LEPs agreed that images lacking bodily diversity had the potential to cause men to feel excluded by resources where they do not feel represented, and can trigger negative comparisons, which may exacerbate EEBIP concerns.

### Phase 3—iterative co-design

The iterative co-design produced three drafts, culminating in the final guidance document (Additional file 1). The first draft, based on the themes reported above, was reviewed by five academic representatives and 10 representatives from healthcare organisations (charities and services). Feedback informed several revisions, including reframing recommendations to emphasise what to do rather than what not to do, and integrating more LEP quotes to evidence the guidance. A second version was then reviewed by three LEPs, who largely agreed with the content but suggested minor changes and the addition of a step-by-step guide to reduce the potential for staff to become overwhelmed, which was subsequently incorporated.

## Discussion

The present study identified priority ideas for the format and content of outreach resources for primarily UK-based men experiencing EEBIP (Phase 1), explored these ideas through lived-experience discussions (Phase 2), and used the findings to inform practical guidance for services (Phase 3). The results reveal both overlap and divergence between existing men’s mental health evidence and the perspectives of LEPs in this study, as discussed below. Notably, whilst not statistically generalisable, the findings of this study highlight considerations—from men with EEBIP—for future EEBIP outreach resources, and these findings may be transferable to wider groups of men [69].

### Similarities with general mental health studies

LEPs called for purpose-driven, lived-experience-based resources featuring relatable men in conversational, non-medicalised formats. These preferences mirror findings in men’s mental health research [37, 39–41, 70]. Considering that this is the first study to gather lived-experience suggestions for EEBIP-specific outreach aimed at men, the consistencies provide further evidence that these suggestions may be broadly effective across different populations of men.

Of note, the LEPs in the present study spoke of moving beyond messages of ‘coping’ (e.g., ‘it’s okay not to be okay’) towards messages of action by suggesting purpose-driven language that aligns with common masculine values. However, although LEPs suggest that these messages may be useful to help engage men, those designing these resources should consider aligning messages with multiple masculinities, including non-traditional modern masculine identities [71–74] in order to reduce the likelihood of reinforcing rigid masculine ideals (e.g., that it is emasculating to experience poor mental health; [75–77]. This consideration of various masculinities promotes the necessity for multiple resources that target different groups of men (as noted in Theme 1), as resources are unable to capture the needs and experiences of all men.

While many of the findings align with existing literature, some appear to be unique suggestions which may be specific to EEBIP help-seeking. These novel findings are discussed below.

### ‘Readiness’

Past studies note that men often delay help-seeking due to limited EEBIP literacy [3, 78], which can prevent recognition of symptoms, hindering help-seeking [79, 80]. Low mental health literacy also contributes to stigma in men, which may reduce help-seeking further [81, 82]. Theme 1 captures the need for organisations to consider men’s EEBIP literacy, feelings of stigma, and attitudes towards help-seeking (i.e., men’s ‘readiness’) when designing outreach resources. While various models of behaviour change may apply to ‘readiness’ (e.g., the stages of change model; [83]), LEPs’ emphasis on EEBIP awareness and help-seeking attitudes suggests outreach should meet men where they are, offering accessible entry points that match differing levels of EEBIP literacy and willingness to seek help.

### Facilitating self-recognition

One factor that may limit men’s EEBIP literacy is the broader societal perception of behaviours commonly associated with EEBIP, such as exercising, which can be both healthy and health-concerning (e.g., those who experience relative energy deficiency in sport; [84]). This creates a unique challenge for public-facing information about EEBIP, compared with other health concerns, as such resources must explicitly account for the cultural framing of these behaviours. As highlighted in Theme 3, LEPs noted that the normalisation of potentially EEBIP-related behaviours/cognitions, particularly within bodybuilding communities, can obscure recognition of underlying problems. Indeed, the bodybuilding community has been a key population of focus for muscle dysmorphia research [85]. Qualitative studies demonstrate how those with muscle dysmorphia often normalise EEBIP-related behaviours, as the behaviours align with personal values and goals [86, 87]. Bodybuilders reportedly share an expectation and normalisation of persistent body dissatisfaction or dysmorphia, reflected within a common phrase from bodybuilding communities: “the day you start lifting is the day you become forever small” [88].

Because of this normalisation, LEPs expressed that resources labelling EEBIP behaviours as disordered can feel adversarial and may cause some men to become guarded against attempts to change their minds. LEPs suggested that public-facing resources should instead aim to facilitate the process of recognising the behaviours as symptoms (i.e., self-realisation). This way, LEPs believe men will feel a greater sense of agency, whilst limiting the likelihood of men experiencing a false sense of health when their specific symptoms are not listed as disordered. Men’s relationship with some behaviours associated with EEBIP can change across time, bringing both negative and positive effects (e.g., biopsychosocial benefits and identity development interspersed with phases of body dysmorphia and consideration of risk behaviours [steroids]; [89, 90]). Encouraging reflection and providing initial self-help options may support men’s agency and help them make informed decisions about seeking support.

### Utilising bodily images

The media’s representation of bodies is often linked to increased EEBIP concerns [91, 92]. BEAT’s (the UK’s largest ED charity) media guidelines [93] advise against using “images of emaciation” (p.5) or bodies “of low weight” (p.15) and promote visuals of “positive experiences and togetherness” (p.16). In Theme 5, LEPs broadly supported this guidance but added insights specific to their experiences as men. LEP6 noted that including severe male presentations, even at low weight, may increase awareness that men can experience extreme EEBIP symptoms and support recognition of men’s EEBIP. LEP4 saw potential in using muscular images, if accompanied by contextual information, to challenge unrealistic ideals and highlight the biopsychosocial risks of pursuing extreme muscularity [94–97].

### Strengths and limitations

This study is the first to explore men’s perspectives on facilitating men’s EEBIP healthcare help-seeking through public-facing resources. Specifically, this study identified LEPs’ preferred formats, content, and considerations for developing EEBIP outreach resources inclusive of men, addressing key gaps in both EEBIP and men’s help-seeking facilitation literature [6, 38].

In terms of limitations, there were several intersectional areas which were not represented in the group (e.g., transgender men, various ethnicities, sexual orientations, ages, EEBIP experiences), and even those that were represented were not always discussed extensively (as noted by LEP6 regarding the lack of discussion of the intricacies of creating resources for gay men). Additionally, LEP5 reflected on the level of EEBIP and mental health literacy within the focus group, in addition to the educational status of many LEPs, which (although not recorded through demographic data) were obvious at times as LEP referred to psychological theory (i.e., the stages of change model) and showed high levels of self-reflection. Together, these reflections from LEPs point out that the mostly white British, educated, and cisgender LEP group is unable to reflect the needs of all men experiencing EEBIP.

Furthermore, although the initial idea generation survey was advertised via multiple channels, one of the primary channels included emails to undergraduate cohorts. This may have influenced the content and format ideas due to selection bias and education levels, which should be considered when reviewing Tables 4 and 5.

A further limitation involves the lead researcher’s role in the NGT’s “clarification and consolidation” phase. While this aligns with the axiological stance of RTA, it may have led to the grouping of distinct ideas and a loss of nuance. Future research might mitigate this by incorporating participant validation or co-analysis.

Future studies should continue to identify further considerations for public-facing resources with diverse men (e.g., different ethnicities, sexual identities, educational backgrounds, socioeconomic status, and EEBIP experiences). These future studies should aim to utilise co-production methods to promote epistemic justice in the identification of further EEBIP outreach resource considerations. Studies may then look to develop resources and test the effectiveness of these considerations on men’s recognition of EEBIP and help-seeking intention and/or behaviours.

## Conclusions

This study contributes to the fields of men’s EEBIP and men’s mental health help-seeking by centring the experiential knowledge of men with lived experience of EEBIP to inform the design of inclusive, public-facing healthcare resources. While previous research has primarily focused on barriers to men’s help-seeking, particularly in general mental health contexts, this study explores potential facilitators of EEBIP help-seeking through a co-designed, lived-experience-led approach.

This study identifies LEPs’ preferred formats and content for public-facing resources, as well as five core themes that may guide inclusive resource design. These findings build on existing evidence on men’s help-seeking and men-inclusive communication by offering EEBIP-specific insights into how healthcare organisations might create materials that can more effectively engage some men.

The study also demonstrates a methodological approach through the integration of NGT and participatory methods as part of an iterative co-design process, utilising lived-experience facilitation and co-facilitation of data collection. This approach enabled exploration of the values, tensions, and practicalities inherent in designing men-inclusive resources. However, the study’s predominantly white, cisgender sample and UK-focused context limit the generalizability of these findings. The recommendations reflect the perspectives of participants in this study and may not represent all men experiencing EEBIP, particularly those from minoritised ethnic groups, transgender men, or men from different cultural contexts.

Further research is required to examine the applicability of these recommendations across diverse groups of men, to test whether resources designed using this guidance actually improve men’s engagement and help-seeking behaviours, and to evaluate how the proposed guidance translates into practice across different organisational settings. Empirical evaluation comparing outcomes before and after implementation of these recommendations would strengthen the evidence base for this approach.

Together with the accompanying guidance document (see Additional file 1), these findings offer a preliminary foundation for healthcare organisations seeking to create public-facing resources that may be more inclusive and responsive to some men experiencing EEBIP. The guidance should be viewed as a starting point that requires iterative refinement based on implementation experience and feedback from diverse communities of men.

## Implementation

This study led to the co-design of the “Outreach resource with men in mind” guidance document. The guidance is included as an Additional file and is also available at MyoMinds.co.uk. The guidance has been shared across social media and through the research team’s wider connections. As noted in the discussion section, the findings from this study—as presented in the resulting guidance—will not generalise to all men; however, the findings and guidance highlight considerations that may be transferable across diverse groups of men [69]. Staff within EEBIP healthcare organisations should look to use the guidance document to inform the co-creation of future resources alongside men with lived experience and/or as a tool to reflect on whether current resources may appear appropriate to men. We hope that the guidance inspires and empowers staff to initiate the co-development of outreach resources with men in mind, leading to new and varied resources for men.

## Supplementary Information

Supplementary Material 1. Outreach resources with men in mind: a guidance document. The co-designed guidance document for healthcare services and charities, developed as a result of the present study. The guidance document presents practical guidance for healthcare services and charities looking to develop public-facing materials that are inclusive to men

## Data Availability

The datasets used and/or analysed during the current study are available from the corresponding author on reasonable request.

## Abbreviations

EEBIP: Eating exercise and/or body image psychopathology
EBIP: Eating and/or body image psychopathology
ED: Eating disorder
BDD: Body dysmorphic disorder
NGT: Nominal group technique
LEP: Lived experience partner
RTA: Reflexive thematic analysis
