# Supplementary material for: “I wanna live in a world where change is possible”: co-designing guidance for inclusive eating, exercise, and body image psychopathology outreach resources for men
Source: J Eat Disord. 2026 Mar 5;14:76. doi: 10.1186/s40337-026-01562-5 (PMC13045136; doi:10.1186/s40337-026-01562-5)
Supplement: Supplementary file 1 — Supplementary Material 1. Outreach resources with men in mind: a guidance document. The co-designed guidance document for healthcare services and charities, developed as a result of the present study. The guidance document presents practical guidance for healthcare services and charities looking to develop public-facing materials that are inclusive to men [file 40337_2026_1562_MOESM1_ESM.pdf]

OUTREACH RESOURCES WITH MEN IN MIND

GUIDANCE FOR HEALTHCARE ORGANISATIONS THAT SUPPORT EATING, EXERCISE, AND/OR BODY IMAGE RELATED CONCERNS.

This guidance was co-created* with men to support all healthcare organisations (NHS, Private, Charities, and Private Practices) hoping to reach men with eating, exercise, and/or body image concerns (e.g., eating disorders, body dysmorphic disorder) via outreach resources.

"Outreach resources" are defined as: *public-facing materials/activities designed to engage people who are not yet accessing support and/or are unfamiliar with the healthcare organisation (e.g., leaflets, videos, events, etc.)*

*(see final page for more detail).

UNIVERSITY of WORCESTER

MyoMinds

First Steps ED
Supporting Every Step of Your Eating Disorder Journey

PRINCIPLES FOR DESIGNING OUTREACH RESOURCES FOR MEN

Use these three principles to review your organisation's current resources or to inform the development of new ones. For those in services: this guidance may be useful for peer support workers, provider collaboratives, or others involved in outreach and service user engagement.

Note: This guidance was co-created with men, but we do not represent all men. The best approach is to work with, and seek feedback from, men local to your organisation (see p.4).

PRINCIPLE 1: RESOURCES SHOULD BE PERSON-CENTRED, WITH AUTHENTIC CONVERSATION WITH MEN AS A FOUNDATION.

Resources should be built from real conversations (e.g., following men's stories and using direct quotes) with diverse men who have lived experience. Conversation can help ground the resource in authentic language that resonates with men.

Note: no single experience captures all men's realities. Creating resources should be a continual process of engagement across diverse groups of men to encompass various experiences.

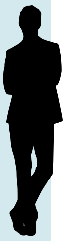

One man said:
"People from different regions, social backgrounds, educational backgrounds. They will all use different language."

PRINCIPLE 2: RESOURCES SHOULD PROVIDE A PURPOSE TO ACCESSING SUPPORT AND POTENTIAL FOR PROGRESSION.

One man said:
"I don't want to be grounded in that [it's okay to not be okay] reality. I want something that's like, you know, 'build resilience'. That's aspirational"

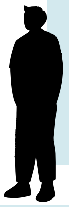

Move beyond messages like "It's okay to not be okay." While these can be comforting, they lack direction. Instead, ask service users what they found helpful about accessing support, and share these men's stories (see Principle 1) to highlight what can be gained.

This principle is not about removing safety or support messages, but adding a sense of purpose. Behaviours like restrictive eating and excessive exercise may be viewed by some as healthy and aspirational. So provide messages that also feel like progress, not just a lifeline for something that may not be seen as a problem yet.

PRINCIPLE 3: RESOURCES SHOULD ENCOURAGE SELF-REALISATION AND REFRAIN FROM LABELLING MEN AS 'UNHEALTHY' OR 'DISORDERED'.

Encourage men to reflect on their experiences through prompts (e.g., "Do you find it hard to stop thinking about food?") or by displaying examples of other men discussing their experiences (see Principle 1) and how support influenced their lives (see Principle 2).

When labelling behaviours as 'disordered', resources may alienate men. Instead, show examples of how the behaviours could be disordered (through men's experiences) and/or directly prompt men to consider how these behaviours may influence their lives.

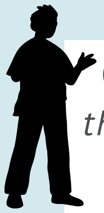

One man said: *"It's that honest reflection of someone's experience. Rather than 'here's a problem, and you might be that person' It's more like 'this is a thing that I was experiencing'"*

CONSIDER THE AUDIENCE

2

Tailor outreach resources to specific groups of men. Resources for 'all men' may not reach any men. When identifying your audience, consider:

- **Demographics** (e.g., race, sexual orientation, socioeconomic background, etc.)
- **Experiences** (e.g., eating/body image concerns, occupational/cultural language/slang, etc.)
- **Contextual setting** (e.g., where they live or spend time in person, as well as digital environments like social media or online communities they engage with).
- **Stage of seeking support -** see example 'stages' below, these are not exhaustive.

STAGES TOWARD SEEKING SUPPORT (WITH EXAMPLE IDEAS)

For men who do not yet recognise the impact of their attitudes and behaviours.

- Develop educational resources for peers/family (e.g., how to approach someone they are worried about, or how to support someone experiencing these concerns).
- Ask organisations that men may already engage with (e.g., gyms, bars) to share/display your outreach resources.
- Ensure resources are easily accessible, passive (e.g., can listen while doing other things), and discreet (e.g. posters in bathrooms, audio that can be played privately).
- Avoid labelling behaviours as 'symptoms' - instead, ask questions (e.g., do you still exercise whilst injured?) and offer information about support available.

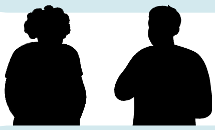

For men who recognise the impact of their attitudes and behaviours but do not know what it is or how to seek support.

- Encourage self-recognition (i.e., of what the symptoms might be and where they could access support) through sharing men's stories of seeking and receiving support.
- Work with local support groups that men may already use (e.g., men's groups) to outline and share the experiences your organisation can help with.
- Provide self-help options for men to try, and links for further support if needed.

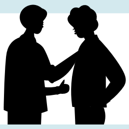

For men who know they could attempt to access support from your organisation, but have not done so.

- Ground the resource in men's stories, using men's words and verbiage (see Principle 1).
- Content that men can share with friends/family (e.g. social media posts) as an initial attempt to seek help, as some men may find this easier than starting a conversation.
- Use messaging that provides purpose and progression (e.g., how can support improve men's lives? What useful skills can men expect to learn?; see Principle 2).
- Provide clear, practical guidance on accessing your organisation (e.g., what to say, who to speak to, what to expect, and what to do if support is not immediately available.)

FORMAT OF OUTREACH RESOURCES (WITH EXAMPLES)

Consider the format of your resource and how this may influence the way the resource can be used. The men in our group suggested these preferred formats:

1st: Podcasts. Long-form conversations that can be divided into short, accessible clips that are easy to share, similar to those featured in the NHS "Keeping Safe Programme".

2nd: Social media posts and campaigns. Can reach wider audiences, are easily shareable, and provide accessible information (e.g., see @connectlypft on Instagram for a service example).

Joint 3rd: Lived experience talks. Authentic conversation is powerful, but be aware that men may feel too stigmatised to attend such events. Online talks can allow for anonymity, which may help engagement.

Joint 3rd: Leaflets and posters. Provide QR codes to more information. Display location is important as men may feel stigmatised if looking at the poster/leaflet around others.

USING IMAGES IN YOUR OUTREACH RESOURCES

Men's discussions regarding images highlighted the importance of specified resources for the target audience (see p.2), as men had differing opinions of what are engaging and appropriate images. **Two points reached consensus:**

1) Men must be represented within the images contained within resources.
2) The images should show the diverse range of men who experience these concerns.

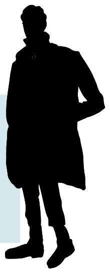

One man said: *"I don't think you just want one specific body type on a poster. I think that's going to be incredibly shaming, and could discourage people from getting help."*

During the discussion of images, men debated several ideas and offered two areas for consideration for organisations to reflect on when developing outreach resources. These may be discussed with the men with lived experience involved in your resource development:

1) Images of bodies may be effective at reaching target audiences, especially by showing diverse body shapes to ensure men feel represented and included. However, consider that images may encourage negative comparisons, so image inclusion should be handled sensitively (e.g., asking men with lived experience to review the images selected).

2) In some cases, sharing images of bodies (even extreme examples) in the context of eating, exercise, or body image concerns may be appropriate as a way to raise awareness about related issues. For example, people are likely to see images of muscular bodies in their everyday lives (e.g., on social media), and these physiques are often labelled as 'healthy' across society. Resources that display these muscular body images alongside thoughtful and nuanced messaging can help raise awareness about eating, exercise, and body image concerns in men and people with different body types.

SUMMARY: HOW TO USE THIS GUIDANCE

To help you and your organisation operationalise this information, we have put together a step-by-step guide to create a resource that follows the suggestions described by men in the previous pages.

Note: Creating resources is complex, and this step-by-step guide is not the only way to design resources in line with the suggestions in this document. This is simply just to help those who may be unsure how to proceed.

STEP 1 - BUILD YOUR TEAM

To create resources inclusive to men, work with men with lived experience to guide the development of resources.

STEP 2 - CONSIDER YOUR AUDIENCE

Who is this resource specifically intended for? The more clearly you define your audience, the better you can tailor the resource to meet their needs. Be sure to consider where they are in their journey toward seeking help (see p.2), as different stages may require different types of information and support.

STEP 3 - CONSIDER FORMAT(S) OF RESOURCES

Each format has its advantages and limitations (see p.3), and should be considered based on the men you are aiming to reach (as identified in Step 2). A single conversation can generate multiple resources—for example, a lived experience talk can be recorded and edited into a podcast series, with quotes or clips repurposed for social media, leaflets, and posters.

STEP 4 - LIVED EXPERIENCE AND CONVERSATION-LED

Build your resource around conversations with a man/men with lived experience. Use direct quotes to ensure an authentic voice, and let these quotes shape the focus and direction of the resource.

STEP 5 - ENCOURAGE SELF-REALISATION

Sharing men's stories may encourage realisation, especially if the story describes attitudes or behaviours that someone may identify with (e.g., "I was terrified to take my top off at the pool"). Additionally, prompting questions may initiate self-reflection from men.

STEP 6 - DEVELOP PURPOSE-FOCUSED INFORMATION

Build information around men's lived experience that speaks to the purpose of accessing healthcare. Detailing, for example, what men can gain from accessing support, and how this support might help them.

STEP 7 - CONSIDER IMAGES

Adding images to some resources may help with engagement. Images of men can help, but consider how you use these images. Be sure to select images with discretion and sensitivity.

OUTREACH RESOURCES WITH MEN IN MIND

CO-DESIGN PROCESS:

1. 41 Men shared their ideas for effective content and format of eating, exercise and body image concern outreach resources via an online survey.
2. Six men with lived experience reviewed the ideas submitted by men and voted for their favourites.
3. The men with lived experience discussed their favourite ideas to explore the underlying principles they believe will help engage men, alongside other considerations.
4. The themes discussed by the lived experience men informed the first draft of this guidance document.
5. The draft guidance was the reviewed by healthcare organisation representatives (n=10), and academic representatives (n=4), to inform edits.
6. The edited draft was reviewed by the lived experience men to inform final edits.

TO ACCESS THE RESEARCH UNDERPINNING THIS WORK:

THANK YOU TO:

The men whose responses and discussions underpin this guidance.

The representatives from healthcare organisations and academia who reviewed this guidance.

RESEARCH TEAM:

George Mycock[a,b], James Downs[c], Christian Edwards[a], Győző Molnár[a], Una Foye[d], Heike Bartel[e], Jess R. Griffiths[f]

(a)University of Worcester, (b)MyoMinds, (c)Lived experience peer researcher, (d)Kings College London, (e)University of Nottingham, (f)South London and Maudsley NHS Trust.

Have you got feedback or questions?

Contact us: myomindsgeorge@gmail.com; c.edwards@worc.ac.uk

UNIVERSITY of WORCESTER

MyoMinds

First Steps ED
Supporting Every Step of Your Eating Disorder Journey
